# Practical Guide for Anticoagulant and Antiplatelet Reversal in Clinical Practice

**DOI:** 10.3390/pharmacy11010034

**Published:** 2023-02-11

**Authors:** Mohammed Aldhaeefi, Hisham A. Badreldin, Faisal Alsuwayyid, Tariq Alqahtani, Omar Alshaya, Majed S. Al Yami, Khalid Bin Saleh, Shmeylan A. Al Harbi, Abdulrahman I. Alshaya

**Affiliations:** 1 Department of Clinical and Administrative Pharmacy Sciences, Howard University College of Pharmacy, Washington, DC 20059, USA; 2 Pharmaceutical Care Services, King Abdulaziz Medical Center, Riyadh 11426, Saudi Arabia; 3 College of Pharmacy, King Saud bin Abdulaziz University for Health Sciences, Riyadh 11426, Saudi Arabia; 4 King Abdullah International Medical Research Center, Riyadh 11426, Saudi Arabia; 5 Department of Pharmaceutical Sciences, King Saud bin Abdulaziz University for Health Sciences, Riyadh 11426, Saudi Arabia

**Keywords:** pharmacist, pharmacy technician, health promotion, health protection, health improvement, interventions

## Abstract

In recent years, anticoagulant and antiplatelet use have increased over the past years for the prevention and treatment of several cardiovascular conditions. Due to the rising use of antithrombotic medications and the complexity of specific clinical cases requiring such therapies, bleeding remains the primary concern among patients using antithrombotics. Direct oral anticoagulants (DOACs) include rivaroxaban, apixaban, edoxaban, and betrixaban. Direct thrombin inhibitors (DTIs) include argatroban, bivalirudin, and dabigatran. DOACs are associated with lower rates of fatal, life-threatening, and significant bleeding risks compared to those of warfarin. The immediate reversal of these agents can be indicated in an emergency setting. Antithrombotic reversal recommendations are still in development. Vitamin K and prothrombin complex concentrate (PCCs) can be used for warfarin reversal. Andexanet alfa and idarucizumab are specific reversal agents for DOACs and DTIs, respectively. Protamine sulfate is the solely approved reversal agent for unfractionated heparin (UFH) and low-molecular-weight heparin (LMWH). However, there are no specific reversal agents for antiplatelets. This article aims to provide a practical guide for clinicians regarding the reversal of anticoagulants and antiplatelets in clinical practice based on the most recent studies.

## 1. Introduction

Anticoagulant use has increased in recent years for the prevention and treatment of several cardiovascular conditions, such as pulmonary embolism (PE), deep vein thrombosis (DVT), atrial fibrillation (AF), mechanical heart valve thrombosis, and acute coronary syndromes [1]. Moreover, the role adopted by antiplatelets as primary or secondary prophylaxis strategies has recently expanded over the past few years to include patients with mild ischemic strokes, in addition to acute coronary syndrome and peripheral artery diseases. This widespread use of anticoagulants and antiplatelets carries with it an avertible risk of major and minor bleeding [2]. Moreover, hospitalizations and emergency department visits due to bleeding induced by anticoagulation have been increasing [2]. This bleeding risk can be significantly exacerbated if there is a compelling indication of combination therapy within specific patients, consisting of an anticoagulant plus antiplatelet or dual antiplatelet therapy [3]. When such drugs are employed as a monotherapy, warfarin and aspirin carry the highest bleeding risk [3].

Warfarin is a vitamin K antagonist that inhibits vitamin K epoxide reductase complex 1 (VKORC1), which reduces the vitamin K-dependent coagulation factors activity. Rivaroxaban, apixaban, and edoxaban exert their anticoagulant effect through Factor Xa inhibition. Antiplatelets are classified into two main categories based on the mechanism of platelet inhibition irreversible inhibition with prasugrel, clopidogrel, and aspirin and reversible inhibition with ticagrelor. UFH and LMWH inactivate thrombin and activated factor Xa through antithrombin (AT)-dependent mechanisms. Fondaparinux is a catalytic factor Xa inhibitor. Argatroban, bivalirudin, and dabigatran are thrombin inhibitors [4,5]. Reversing the anticoagulant and antiplatelet effect is fundamental to preventing fatalities from major life-threatening bleeding events [3]. A deep understanding of the magnitude of antithrombotic drug influences on differing clotting tests is essential in monitoring the drug reversal’s success (Table 1) [3].

**Figure 1:**
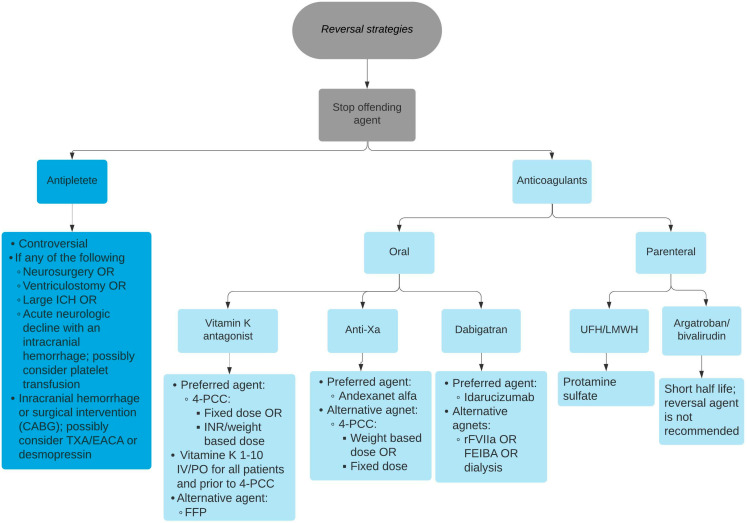
Reversal strategies of different oral and parenteral anticoagulant and antiplatelet agents. ICH: Intracerebral hemorrhage. CABG: Coronary artery bypass graft surgery. TXA: Tranexamic acid. EACA: Aminocaproic acid. 4F-PCC: Four-factor Prothrombin complex concentrates. INR: International normalized ratio. IV: Intravenous. PO: Per os. FFP: Fresh frozen plasma. rFVIIa: Recombinant factor VIIa. UFH: Unfractionated heparin LMWH: Low-molecular-weight heparin.

The currently used clotting tests have several limitations, for example, they can be unreliable in critical illness, have a prolonged timeframe for exerting the pharmacological function, and they are unable to measure the platelet function and activity of the clotting cascade [6]. Viscoelastography (VE) including thromboelastography (TEG) and rotational thromboelastometry (ROTEM) are currently widely used in clinical practice to overcome these impractical issues of the currently use clotting tests [6]. VE includes thromboelastography (TEG) and rotational thromboelastometry (ROTEM) [6]. Both techniques have the ability to run a qualitative and quantitative coagulopathy assessment and measure the degree of fibrinolysis [6]. VE can facilitate selecting a directed approach to antithrombotic reversal strategies [6]. However, VE utilizes only in vitro blood coagulation evaluation, which does not account for other associated factors such as the blood flow properties, injured vessel size, and vessel wall anatomy, which determine the membrane-bound pro- and anticoagulation factors. Furthermore, VE requires additional resources and requires expert personnel to run and interpret all the analyses. The objective of this article is to provide a practical guide for clinicians regarding reversing anticoagulants and antiplatelets in clinical practice based on the most recent studies (Figure 1).

## 2. Specific Reversal Strategies

### 2.1. Antiplatelet Drugs

Platelet aggregation recovery time (from the patient’s last dose) varies among the antiplatelet drugs. Typically, it is estimated at approximately 4–5 days for aspirin, clopidogrel, and ticagrelor. However, it is approximately seven days for prasugrel [7]. Although the data are limited and controversial, several reversal agents have been reported to be used in the setting of major and minor bleeds while the patients were on antiplatelet therapy. Such agents include desmopressin (DDAVP), antifibrinolytic therapy, and platelet transfusion. The dosing details are summarized in Table 2.

#### 2.1.1. Desmopressin (DDAVP)

DDAVP pharmacology involves the triggered release of Factor VIII and the von Willebrand factor, leading to secondary improvements in the platelet adhesion to the endothelial defects [8]. It is a medication with a relatively safe drug profile. However, DDAVP could lead to edema after repeated dosing [8]. The clinical utilization of DDAVP for antiplatelet reversal is controversial. A small retrospective study (*n* = 14) focusing on patients with an intracerebral hemorrhage (ICH) and known aspirin use revealed that DDAVP resulted in a statistically significant reduction of the Platelet Function Analyzer, epinephrine (from 192 ± 18 to 124 ± 15 s) [9]. In addition, the vWF antigen activity increased from 242 ± 96% to 289 ± 103% (*p* = 0.004), and only two patients developed hematoma/s [9]. A larger analysis included 55 patients receiving DDAVP, highlighting that DDAVP significantly lowered the incidence of ICH expansion (10.9% vs. 36.2%) [10]. Similar findings were recognized among traumatic brain injury (TBI) patients [11]. The patients had significantly reduced chest tube and total blood loss when DDAVP was used prior to coronary artery bypass grafting [12].

#### 2.1.2. Platelet Transfusion

Supplementing the circulatory system with additional and uninhibited platelets through platelet transfusion has a theoretical benefit for antiplatelet reversal [13]. Platelet transfusion carries the risks of volume overload, anaphylactic reactions, hemolytic adverse reactions, transfusion-related lung injury, and the possibility of infections [13]. The seminal PATCH trial reported that the odds of death or dependence at three months were statistically significantly reduced within the platelet transfusion group compared to those of the control group [14]. However, this was mostly observed in the non-surgical patients. In addition, severe adverse events, including ICH expansion or urinary/pulmonary infections, were more common among the platelet transfusion patients [14]. Similarly, a large retrospective analysis found that non-traumatic ICH patients treated with platelet transfusions had a higher risk of reduction the risks of surgery, disability, and death [15]. Conversely, platelet transfusion within 12 h of symptom onset among patients with ICH was associated with a reduced hematoma expansion and a lower degree disability at three months in a small observational study [16]. Lastly, a large retrospective analysis found that TBI patients who received a platelet transfusion had a statistically significant lower progression rate, a decreased rate of neurosurgical intervention, and reduced odds of being discharged to a skilled nursing facility [17].

#### 2.1.3. Tranexamic Acid (TXA) and Aminocaproic Acid (EACA)

TXA and EACA work as antifibrinolytic therapy by inhibiting the lysine binding site of plasminogen and stabilizing the preformed fibrin meshwork, preventing the conversion of plasminogen into plasmin (Table 2) [18]. Two major prospective trials evaluated antiplatelet reversal in cardiac surgery patients using TXA (bolus dose followed by a maintenance dose) [19,20]. The d strategy was a non-weight-based one, and 1 g bolus was administered intravenously (IV), followed by 200 mg/h as a continuous intravenous infusion (CIVI) and a single 2 g IV bolus dose, respectively [19,20]. The first study reported that the patients within the TXA group received a significantly lower level of packed red blood cell (pRBC) transfusions [19]. The second study did not investigate any clinical outcomes; however, it reported a statistically significant increase in the ADP-induced platelet aggregation among the TXA patients [20]. Conversely, three studies utilized TXA weight-based dosing for antiplatelet reversal in cardiac surgery patients, and 10 mg/Kg IV bolus and 10 mg/Kg CIVI maintenance doses, a single dose of 10 mg/Kg IV bolus, and a single dose of 30 mg/kg IV bolus were administered, respectively [21,22,23]. All three studies reported statistically significant blood loss reduction with TXA use [21,22,23]. A very limited dataset is available to support the use of EACA for antiplatelet reversal. A small prospective study found a statically significant eight-fold increase in deep venous thrombosis with the EACA administration [24]. Moreover, a non-significant reduction of mortality due to a hemorrhage was found among the patients receiving EACA [24].

### 2.2. Warfarin

Halting warfarin as a sole strategy can be sufficient in asymptomatic patients with an elevated INR and a low hemorrhagic risk [25]. If pharmacological intervention is required, several agents can be used, including vitamin K, PCCs, and fresh frozen plasma (FFP) [25].

#### 2.2.1. Vitamin K (Phytonadione)

Exogenous vitamin K can continue to be reduced and converted into the active form (KH2), consequently resulting in functional clotting factors, despite the recent warfarin administration (dosing summarized in Table 2) [25]. Anaphylactic reactions and temporary warfarin resistance have been reported with vitamin K use [25]. IV and per-oral (PO) vitamin K has a similar effect on INR at 24 h. However, IV vitamin K has a more rapid INR lowering effect in comparison to that of PO vitamin K [26]. Although IV vitamin K was more rapid in reducing INR, all the patients had INR > 4 at 4 h following IV and PO vitamin K [26]. Another study found similar outcomes as only 50% of patients had INR < 2 at 24 h when IV vitamin K was used as a monotherapy [27]. Thus, combination therapy should be implemented if the immediate correction of the coagulopathy is required. The lack of thromboembolic risk following vitamin K administration renders it a safe option, especially when the patient is stable [25].

#### 2.2.2. Prothrombin Complex Concentrate (PCC)

PCC contains clotting factors that are isolated from the plasma sample [5]. These clotting factors are 25-fold more concentrated than blood is [5]. PCC dosing is based on factor IX and can be divided into activated versus non-activated ones, in addition to the percentage of clotting factors included to either three-factor PCC or four-factor PCC [25]. PCC can be dosed based on two common strategies, with either fixed or INR-driven PCC dosing, as summarized in Table 2 [25]. A clinical hemostatic was found within 40/44 (93%) patients when PCC was dosed based on INR in addition to vitamin K [28]. Moreover, normal (or in the proximity of normal) concentrations of all four coagulation factors (FIX, FII, FVII, and FX) continued to be found throughout the 48 h observation period, and thromboembolic complications were found only in two (4.5%) patients [28]. Another study utilizing INR-driven dosing found that PCC patients had a statistically significant reduction of the post-treatment INR (2.3 vs. 1.4) [29]. Two randomized clinical trials comparing PCC + vitamin K versus fresh frozen plasma (FFP) found that the combination therapy resulted in statistically significant hemostasis and rapid INR reduction [30,31]. PCC use was associated with a risk of thrombosis and heparin-induced thrombocytopenia [5]. In comparison to FFP, PCC is supplied in a more reduced diluted volume for administration in comparison to that of FFP (PCC: ~20 mLs vs. FFP 30 mL/Kg) and carries a lower risk of volume overload, as it is infused within 15–30 min. Additionally, PCC does not require any thawing time, which is unlike FFP [5,25].

#### 2.2.3. Fresh Frozen Plasma (FFP)

FFP contains all the coagulation factors, including factors II, VII, IX, and X, in a diluted, inactive form [25]. Additionally, it contains fibrinogen and platelets [25]. FFP is a reasonable coagulation factor replacement as an alternative to PCC in case of life-threatening hemorrhage due to its availability. However, FFP use carries the risk of infections and volume overload (1 unit of FFP has a volume of 250 mL) [25]. Consequently, in patients experiencing life-threatening hemorrhage and concomitant warfarin use, PCC can be preferable to FFP. A major clinical trial demonstrated that INR was not corrected in 12 patients who received FFP, while 28 out of 29 patients receiving PCC had correct INR values [32]. Similar findings were revealed in patients undergoing cardiopulmonary bypass surgery and other cardiac procedures [33].

### 2.3. Direct Oral Anticoagulants (DOACs)

Halting anticoagulant therapy as an initial step should be performed after the confirmation of a hemorrhagic event. Andexanet alfa and PCC are commonly used for DOAC reversal [34].

#### 2.3.1. Coagulation Factor Xa Recombinant, Inactivated-Zhzo (Andexanet Alfa)

Andexanet alfa is a recombinant, modified, human factor Xa protein and acts through a competitive binding mechanism, with specificity for anti-Xa agents, eventually restoring factor Xa activity and reversing the anticoagulant effect accordingly [34]. It is approved for use for the reversal of apixaban and rivaroxaban-based anticoagulant therapies in the setting of life-threatening or uncontrolled major bleeds (Table 2) [34]. The typical adverse effects reported with andexanet alfa include hot flushes pyrexia, thromboembolism, myocardial infarction, and ischemic stroke [34]. The approval of andexanet alfa was based on the results of two major randomized clinical trials, ANNEXA-A and ANNEXA-R [35]. Five mg apixaban, administered twice daily (ANNEXA-A), or 20 mg rivaroxaban, administered once daily (ANNEXA-R), were administered to all the healthy volunteers. Andexanet alfa reduced the anti-factor Xa activity within 2–5 min by 94% and 92% following an IV bolus of ANNEXA-A and ANNEXA-R, respectively [35]. The active medication level rebound was detected at 4 h post-administration with apixaban and rivaroxaban [35]. ANNEXA-4 included patients who were treated with apixaban, rivaroxaban, or LMWH within the previous 18 h [36]. Excellent/good hemostasis was identified in 204/249 (82%) of this patient’s cohort, with mortality occurring in 49 (20%) patients 30 day later and thrombotic events identified in 34 (14%) patients with this study [36].

#### 2.3.2. Prothrombin Complex Concentrate (PCC)

Although andexanet alfa is preferred, based on the guidelines recommendations, the role of PCCs in DOAC reversal has been evaluated, and multiple studies have demonstrated its potential role as a DOAC reversal strategy [37,38,39]. Specifically, 4F-PCC was intensively scrutinized for the reversal of rivaroxaban/apixaban therapy reversal, with differing dosing strategies employing fixed and weight-based approaches [37,38,39]. The median dose implemented in such studies had a range of 25–50 units/Kg, and the thromboembolic event rates were reported at a rate of 2–11% [37,38,39].

### 2.4. Unfractionated Heparin (UFH) and Low-Molecular-Weight Heparin (LMWH)

Protamine sulfate entirely reverses the action of UFH, though it only reverses 50% of the LMWH therapeutic effects [40]. Approximately 1 mg of protamine sulfate is required to neutralize 100 units of heparin [40]. The protamine dose varies depending on the selected route of administration for heparin therapy, together with time elapsed since the most recent administration [40,41]. It is recommended to be administered at 1 mg/100 units of IV UFH when it is applied within 2–4 h from the last UFH exposure, with a maximum protamine dose of 50 mg [40,41]. When protamine is used for LMWH reversal, the dose is typically 1 mg of protamine/1 mg of LMWH if the most recent LMWH administration was under 8 h and 0.5 mg of protamine/1 mg of LMWH if the most recent LMWH administration was over 8 h [40]. The typically reported adverse effects of protamine to include a significant anaphylactic response, possibly leading to hemodynamic instability [41]. It is important to avoid protamine overdosing, as this could lead to secondary coagulopathies, followed by impaired platelet function or the inhibition of blood factors [40,41]. Patients with documented fish or insulin-neutral protamine hagedorn insulin (NPH) allergies or patients have undergone a vasectomy should not receive protamine [40,41]. Based on limited clinical data, rFVIIa and activated PCC can partially reverse fondaparinux action [42,43,44].

### 2.5. Direct Thrombin Inhibitors (Argatroban, Bivalirudin, and Dabigatran)

Idarucizumab has been approved for dabigatran therapy reversal [45]. The REVERE-AD trial evaluated Idarucizumab efficacy when 5 mg was administered to 503 patients treated with dabigatran and experiencing bleeding [45]. Coagulation assays revealed a reduction of 50% from the baseline. A total of 68% (134/203) of the patients had bleeding cessation within 24 h, with a median time of 2.5 h [45]. No specific reversal agent could be used for bivalirudin and argatroban [43]. However, one ex vivo study found rFVIIa to significantly reduce PTT more than the placebo did, and they used anticoagulation as measured by TEG [43]. Another study examined the reversal capacity of idarucizumab in the setting of bivalirudin therapy and concluded that idarucizumab is highly specific solely for dabigatran, and consequently, failed to reverse bivalirudin activity [46].

## 3. Future Directions

Hemorrhagic events remain major complication in oral antithrombotic therapeutics. Given the elevated fatality rate of life-threatening bleeding events associated with antithrombotic drug therapies and the rising number of patients using antithrombotics, a few emerging reversal agents are currently being developed. Ciraparantag (Aripazine/PER-997) is a small, water-soluble, synthetic cation that binds to DOACs, UFH, and LMWH through non-covalent hydrogen bonding and electrostatic interactions [47]. PER-977 was found to reduce bleeding within 30 min of administration following rivaroxaban, apixaban, edoxaban, and dabigatran overdoses when it was evaluated in rat animal models [47]. A 300 mg PER-977 IV bolus was able to normalize the whole blood clotting time within 10–30 min, and the effect was sustained for over 24 h when it was administered to volunteers who were either untreated or pretreated with 60 mg of edoxaban within a pharmacokinetic/pharmacodynamic dose-escalation study (100–300 mg) [48]. Two major studies (NCT02207257 and NCT01826266) are expected to provide more insight into the dosing, efficacy, and safety of PER-977 in the near future.

## 4. Conclusions

Anticoagulant and antiplatelet use is on the rise for the treatment and prevention of several cardiovascular diseases. Life-threatening bleeding remains a major adverse event of anticoagulants and antiplatelets. The immediate reversal of these agents if life- threatening bleeding presents is indicated in an emergency setting. The anticoagulation and antiplatelet reversal strategies and agents will continue to evolve.

## Figures and Tables

**Table 1:** Effects of anticoagulants on various clotting tests.

Anticoagulant	aPTT	PT/INR	Elimination Half-Life	Effect of Dialysis
Warfarin	Increase or neutral	Increase	7 days	Not dialyzable
Rivaroxaban	Increase or neutral	Increase or neutral	5–9 h	Not dialyzable
Apixaban	Increase or neutral	Increase or neutral	12 h	Poorly dialyzable
Edoxaban	Neutral	Increase or neutral	10–14 h	Not dialyzable
Betrixaban	Increase	Increase	19–27 h	Not dialyzable
Argatroban	Increase	Increase	30–50 min	Poorly dialyzable
Dabigatran	Increase	Increase or neutral	12–17 h	Dialyzable
Bivalirudin	Increase	Increase or neutral	20–25 min	Poorly dialyzable
IV UFH	Increase	Neutral	30 min	Not dialyzable
LMWH	Increase or neutral	Neutral	4.5–5 h	Not dialyzable

IV: Intravenous. UFH: Unfractionated heparin. LMWH: Low-molecular-weight heparin. aPTT: Activated partial thromboplastin clotting time. PT: Prothrombin time. INR: International normalized ratio.

**Table 2:** Reversal agents dosing strategies.

Targeted Medication	Suggested Reversal Agent	Suggested Reversal Agent Dose	Adverse Reactions
Anti-platelets: Aspirin; Clopidogrel; Prasugrel; Ticagrelor.	DDAVP	0.3–0.4 mcg/kg/dose	Fluid retention Pulmonary edema
	Platelet transfusion	Up to a single apheresis unit or equivalent. Greater doses are not more effective, and lower doses equal to one-half of a standard apheresis unit are equally effective	Volume overload Anaphylactic reactions Hemolytic reactions Transfusion-related Lung injury Infections
	TXA	Loading dose (IV bolus): 1 g, 100 mg/kg or 10 mg/kg Maintenance dose: 30 mg/kg, 200 mg/h, 10 mg/kg, 50 mg/kg or 10 mg/kg	Anaphylactic reactions Thromboembolic risk Visual disturbances
	EACA	Loading dose (IV bolus): 4 g Maintenance dose (CIVI): 1 g/h, with a maximum infusion of 4 h	Anaphylactic reactions Thromboembolic risk Visual disturbances
Warfarin	Vitamin K	Minor bleed: 2–5 mg PO/IV Major bleed: 5–10 mg IV	Anaphylactic reactions Temporary warfarin resistance
	4F-PCC	- Fixed dose: Non-intracranial hemorrhage: 1000 units Intracranial hemorrhage: 1500–2000 units INR and weight-driven dose: INR 2–<4 = 25 units/kg INR 4–6 = 35 units/kg INR >6 = 50 units/kg	HIT Thromboembolic risk
	FFP	10–30 mL/kg (1-unit FFP has a volume of 250 mL)	Infections Volume overload (1-unit FFP has a volume of 250 mL)
Anti-Xa: Rivaroxaban; Apixaban; Edoxaban.	4F-PCC	25–50 units/kg based on actual body weight	HIT Thromboembolic risk
	Andexanet alfa	Last dose within <8 h or time is unknown for rivaroxaban ≤ 10 mg and apixaban ≤ 5 mg or when last dose within >8 h with any dose given: Low dose: 400 mg at a target rate of 30 mg/min followed by 4 mg/min for up to 120 min (480 mg) Last dose within <8 h or time is unknown for rivaroxaban > 10 mg and apixaban > 5 mg: High dose: 800 mg at a target rate of 30 mg/min followed by 8 mg/min for up to 120 min (960 mg)	Rebound anti-Xa within 2 h of infusion completion Thromboembolic risk (myocardial infarction and ischemic stroke) Flushing Fever
Direct thrombin inhibitors: Dabigatran	4F-PCC	25–50 units/kg based on actual body weight	HIT Thromboembolic risk
	Idarucizumab	5 g IV bolus (two separate doses of 2.5 g diluted in 50 mL vials)	Thromboembolic risk
UFH and LMWH	Protamine sulfate	For UFH reversal: 1 mg for every 100 units when used within 2–4 h from the last UFH exposure For LMWH reversal: 1 mg for each 1 mg administered within the prior eight hours	Anaphylactic reactions
Direct thrombin inhibitors: o Argatroban; o Bivalirudin; o Fondaparinux.	rFVIIa	70–90 mcg/kg	Anaphylactic reactions

DDAVP: Desmopressin. TXA: Tranexamic acid. EACA: Aminocaproic acid. 4F-PCC: Four-factor Prothrombin complex concentrates. HIT: Heparin-induced thrombocytopenia. FFP: Fresh frozen plasma. UFH: Unfractionated heparin. LMWH: Low-molecular-weight heparin. rFVIIa: Recombinant factor VIIa.

## Data Availability

No new data were created or analyzed in this study. Data sharing is not applicable to this article.
